# Altered right ventricular papillary muscle position and orientation in patients with dilated left ventricles

**DOI:** 10.1186/1532-429X-11-S1-P71

**Published:** 2009-01-28

**Authors:** Erin M Spinner, Kartik Sundareswaran, Lakshimi Prasad Dasi, Vinod H Thourani, John Oshinski, Ajit P Yoganathan

**Affiliations:** 1 Georgia Institute of Technology, Atlanta, GA USA; 2 Emory University, Atlanta, GA USA

**Keywords:** Right Ventricle, Papillary Muscle, Tricuspid Regurgitation, Congestive Heart Failure Patient, Mitral Valve Regurgitation

## Introduction

Displacement of papillary muscle (PM) positions in the right ventricle (RV) may be an important factor in inducing tricuspid regurgitation (TR). We hypothesize that left ventricular (LV) dilation is one possible mechanism for RV PM displacement; given the adjacent and proximal relative positions of the two ventricles.

## Purpose

In this study, we identify and compare the position of RV PMs in normal subjects and patients with LV dilation to quantitatively establish RV PM positions and the influence of LV dilation on RV PM displacement.

## Methods

**Figure 1:**
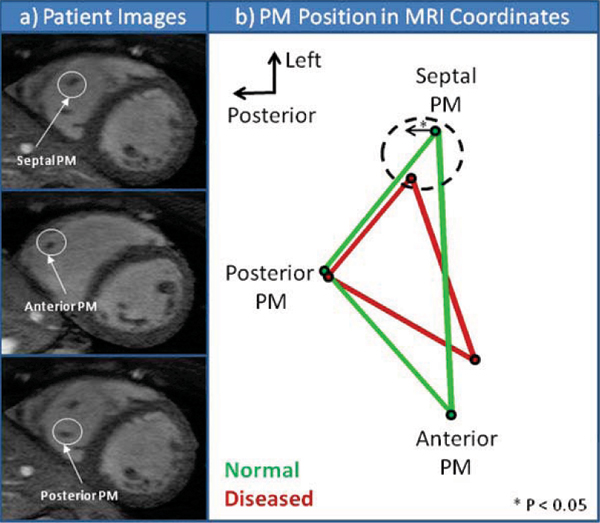
Figure 1

7 congestive heart failure patients with mild to severe mitral valve regurgitation and a dilated left ventricle (LVEDV 234.7 ± 70.8 ml) were recruited at Emory University Hospital. 6 normal subjects were used as a control in this study. Scans were acquired on a Philips Intera CV 1.5 T system using a cardiac coil. The MRI scan to assess PM position was a high resolution, navigator-echo gated 'whole heart' sequence acquired in the short axis orientation. Pixel size ranged from 0.53 – 0.70 mm, slice thickness ranged from 1.5 – 2.0 mm, and echo time ranged from 1.3 – 2.1 ms. Approximately 120 slices were acquired at mid-diastole to cover the entire LV and RV. Papillary muscle tip position was manually identified in the various images by identifying the regions of low intensity (PM) among regions of high intensity (blood) within the right ventricle (RV) (Fig 1a). Coordinates were recorded for all three papillary muscles, septal, posterior and anterior for all subjects. All images were registered to the MRI coordinate system for the purpose of position and orientation comparison. The centroid of the three PM coordinates was used as the reference point (Fig 1b). All distances were normalized by ascending aortic diameter to minimize patient variability. Values for directional distances from the centroid (x, y, z) where calculated for each PM as well as, area of the triangle formed by the three PMs, distance of each triangle side length, and distance from each PM to the centroid. Values for normal subjects and dilated LV patients were then analyzed for statistical significance using parametric unpaired t-test.

## Results

Significant (p < 0.05) posterior displacement of XYZ mm/aortic size of the septal PM from its normal position was observed (Dilated 0.029 ± 0.04 mm/aortic diameter, Normal 0.144 ± 0.03 mm/aortic diameter). More specifically the septal PM significantly displaced from the septal wall (Dilated 0.38 ± 0.04 mm/aortic diameter, Normal 0.61 ± 0.09 mm/aortic diameter). The PM position in normal patients forms a symmetric triangle with sides of similar lengths. For the first time, specific location of all three RV PMs is reported for normal and dilated LV patients (Table 1).

**Table 1:** Numerical measurements of PMs for normal and dilated LV patients

	Normal Average	Normal Standard Error	Dilated LV Average	Dilated LV Standard Error	T-Test P value (* p < 0.05)
**Anterior PM**					
X dist	0.61	0.09	0.38	0.04	0.06
Y dist	0.20	0.04	0.30	0.04	0.11
Z dist	0.20	0.05	0.15	0.03	0.44
**Posterior PM**					
X dist	0.00	0.07	0.03	0.06	0.76
Y dist	-0.35	0.04	-0.33	0.06	0.83
Z dist	0.65	0.03	0.62	0.03	0.48
**Septal PM**					
X dist	-0.61	0.08	-0.41	0.07	0.09
Y dist	0.14	0.03	0.03	0.04	0.03 *
Z dist	0.85	0.07	0.74	0.05	0.24
Dist Ant PM Dist Post PM	0.69 0.76	0.08 0.04	0.49 0.65	0.06 0.10	0.07 0.32
Dist Sept PM	1.07	0.08	0.76	0.11	0.05*
Triangle Area	0.13	0.02	0.09	0.02	0.23
Sept – Ant PM dist	1.40	0.15	1.04	0.08	1.08
Sept – Post PM dist	0.84	0.10	0.63	0.11	0.19
Post – Ant PM dist	0.99	0.07	0.90	0.07	0.35

## Conclusion

Dilatation of the LV has an impact on PMs in the RV. This may have implications in the development of tricuspid valve disease. This also demonstrates that diseases on the left side of the heart also affect the right side of the heart.

